# The cost of research: Lasting effects of capture, surgery and muscle biopsy on brown bear (*Ursus arctos*) movement and physiology

**DOI:** 10.1017/awf.2023.95

**Published:** 2023-11-21

**Authors:** Alexandra Thiel, Anne G Hertel, Sylvain Giroud, Andrea Friebe, Boris Fuchs, Jonas Kindberg, Anne Randi Græsli, Jon M Arnemo, Alina L Evans

**Affiliations:** 1 Department of Forestry and Wildlife Management, Faculty of Applied Ecology and Biotechnology, Inland Norway University of Applied Sciences, Koppang, Norway; 2 Behavioural Ecology, Department of Biology, Ludwig-Maximilians University of Munich, Planegg-Martinsried, Germany; 3 Research Institute of Wildlife Ecology, Department of Interdisciplinary Life Sciences, University of Veterinary Medicine, Vienna, Austria; 4 Norwegian Institute for Nature Research, Trondheim, Norway; 5 Department of Wildlife, Fish and Environmental Studies, Swedish University of Agricultural Sciences, Umeå, Sweden; 6 Energetics Lab, Department of Biology, Northern Michigan University, Marquette, MI, USA

**Keywords:** animal welfare, body temperature, capture effects, ecophysiology, hibernation, movement

## Abstract

Animal models are a key component of translational medicine, helping transfer scientific findings into practical applications for human health. A fundamental principle of research ethics involves weighing the benefits of the research to society against the burden imposed on the animals used for scientific purposes. The utilisation of wild animals for research requires evaluation of the effects of capture and invasive sampling. Determining the severity and duration of these interventions on the animal’s physiology and behaviour allows for refining study methodology and for excluding or accounting for biased data. In this study, 39 Scandinavian brown bears (*Ursus arctos*) captured either while hibernating in winter or via helicopter in summer and that underwent surgery as part of a human health project had their movement, body temperature and timing of onset of hibernation compared with those of 14 control bears that had not been captured during the same period. Bears captured in winter and summer showed decreased movement from den exit until late summer, compared to those in the control group. Bears captured in summer showed reduced movement and body temperature for at least, respectively, 14 and 3 days, with an 11% decrease in hourly distance, compared to pre-capture levels, but did not differ in the timing of hibernation onset. We reveal that brown bear behaviour and physiology can be altered in response to capture and surgery for days to months, post-capture. This has broad implications for the conclusions of wildlife studies that rely upon invasive sampling.

## Introduction

A utilitarian approach to research ethics entails weighing the consequences for the research animal against the benefits of the research to society. Consequences for the animals might include compromised welfare, pain or distress or reduction in survival rates or reproductive success, while benefits are evaluated based on the value of scientific knowledge sought, which can have implications for, e.g. humans (ASAB Ethical Committee/ABS Animal Care Committee 2023). In the field of translational medicine, animal models are used to help understand mechanisms behind diseases and for developing and testing therapies for prevention and treatment in humans. For decades, rodents have been considered the model organisms of choice to study human diseases (Rosenthal & Brown 2007). However, many attempts to translate findings from the laboratory into human medicine fail (Hackam & Redelmeier 2006; Fröbert *et al.* 2020). One of the reasons might be that laboratory animal strains used in medical research are selectively bred to minimise individual variation as much as possible (Sikes & Gannon 2011). Yet variation is an essential part of nature and solutions derived from nature might provide useful tools to mitigate the effects of a changing climate on planetary and human health (Stenvinkel *et al.* 2021).

Despite earlier beliefs that carnivore research has little potential to contribute to human health (Gittleman 2013), Fröbert *et al.* (2020) suggested the use of free-ranging animals as an alternative approach in the field of translational medicine, especially for studying diseases caused by a sedentary lifestyle. The authors point to the brown bear (*Ursus arctos*) as a suitable model species since its seasonal lifestyle results in dramatic fluctuations in activity level and bodyweight (Fröbert *et al.* 2020), mimicking sedentary lifestyle-related conditions in humans, such as physical inactivity and obesity. To unravel the mechanisms allowing brown bears to transition from an obese state in summer to immobility coupled with weight loss during winter hibernation, without experiencing any of the pathophysiological conditions to which humans would typically succumb, is of great interest for interdisciplinary researchers aiming to translate findings from the brown bear into human medicine (see Stenvinkel *et al.* 2013; Chanon *et al.* 2018; Chazarin *et al.* 2019; Luu *et al.* 2020; Ebert *et al.* 2020; Giroud *et al.* 2021; Thienel *et al.* 2023). A recent study derived from this research project shed light upon the thrombo-protective mechanism seen in hibernating bears, providing fundamental insights that can guide the development of a treatment plan for venous thromboembolism in humans (Thienel *et al.* 2023). Serum derived from hibernating bears has revealed the potential for developing new tools to fight human muscle atrophy and related metabolic disorders (Chanon *et al.* 2018) and Chazarin *et al.* (2019) have shown that reduced oxidative stress underlies the resistance to skeletal muscle atrophy in hibernating bears, providing therapeutic targets of human muscle atrophy. The Scandinavian brown bear research project (SBBRP) facilitates this research by capturing free-ranging brown bears during their hibernation phase in winter and active period in summer, enabling sample collection, including muscle tissues.

Regardless of the interdisciplinary effort of medical doctors, physiologists, veterinarians and ecologists working together on this translational study, ultimately aiming to improve human health, the well-being of the animals needs to be ensured. Moreover, consideration must be given to the differing and perhaps heightened consequences faced by wild animals compared to those in captivity (Gittleman 2013). Capturing, anaesthetising and handling wildlife alone are stressful procedures with potentially long-lasting effects on the animals’ behaviour and physiology (Wilson & McMahon 2006). In wildlife research, physical capture is one of the most critical aspects as regards animal welfare since it has the potential to directly or indirectly affect the animals’ welfare state (Soulsbury *et al.* 2020). Additionally, equipping animals with biologgers and/or conducting surgery and invasive sampling, such as muscle biopsies, on a wild species needs to be well justified as this type of procedure carries the risk of inflicting pain, not to mention the various complications that may ensue (Chinnadurai *et al.* 2016; Arnemo *et al.* 2018). Evaluating the effects of capture, handling and invasive sampling procedures is a central part of conducting ethical wildlife research and facilitates refinement of methods to ensure the welfare of the animals being studied. Nevertheless, the precise magnitude and duration of stress induced by handling and sampling procedures in wildlife remain uncertain for the majority of species and specific handling protocols, highlighting the necessity of investigating this crucial aspect of wildlife studies, are required (Soulsbury *et al.* 2020). In reporting the impact a study has on the animals as a standard approach to wildlife research (Wilson *et al.* 2019) researchers are endorsing the shift in researcher culture towards a greater attention to animal care and welfare (Field *et al.* 2019). After all, a key point of the 3Rs principles (replacement, reduction, and refinement) in animal research is refinement and improvement of methods, including minimisation of pain and distress (Lindsjö *et al.* 2016).

In contrast to the controlled environments of animal clinics and laboratories, field conditions are rarely ideal, despite all efforts to conduct the capture as efficiently and aseptically as possible and minimise stress and disturbance to the animal (Cattet 2013). Capture-related mortalities are rare and estimated at 0.5% for Scandinavian brown bears, including those having undergone surgery (Arnemo *et al.* 2013; 1,824 brown bears captured from 1984–2013 with 16 mortalities). However, while ensuring low capture-related mortality rates is important, using this as the stand-alone measure to evaluate animal welfare is not sufficient and other factors need to be taken into account in safeguarding the well-being of animals during captures. Remote monitoring of physiological parameters and behaviour through biologging is one common method of evaluating immediate and persistent effects of physical capture on wildlife. Brown bears captured during their hibernation period were physiologically affected, i.e. increased body temperature and heart rate for at least three weeks post-capture and delayed emergence from their dens for up to nine days (Evans *et al.* 2016b). Cattet *et al.* (2008) showed that movement rates of American black (*Ursus americanus*) and grizzly (*Ursus arctos horribilis*) bears are reduced for up to six weeks following a capture event with additional long-term effects on body condition. A study on polar bears (*Ursus maritimus)* evaluated activity levels and body temperature during helicopter captures and revealed that ambient temperature and the length of helicopter operations influenced the stress response of the bears but that activity levels and body temperature during the captures were comparable to natural behaviour (Whiteman *et al.* 2022). Moreover, helicopter captures may also temporarily disrupt movement and social organisation in wolf (*Canis lupus*) packs by affecting cohesion of pack members (Nordli *et al.* 2023).

It is not purely the immediate, short-term effects that are important to evaluate, but also potential long-term effects since long-term behavioural and physiological measures beyond the focus of the study in question are often neglected, meaning the true impact of deployed devices or sampling procedure may be unknown (Soulsbury *et al.* 2020). A study on King penguins (*Aptenodytes patagonicus*) found that over a period of ten years flipper-banding affected major life-history traits in the marked individuals which ultimately brought into question the legitimacy of conclusions drawn from such individuals (Saraux *et al.* 2011). A study on beavers (*Castor fiber*) found negative effects on several fitness-related parameters in relation to repeated captures (Mortensen & Rosell 2020). On the other hand, a study on polar bears, evaluating the extent to which capture, collaring and handling influenced body mass, body condition and reproduction over six months after capture and found no negative capture-related effects (Rode *et al.* 2014). In fact, polar bear subpopulations equipped with satellite telemetry devices provided valuable information about survival rates, density estimations and effects of sea-ice loss as well as informing management regarding appropriated regulatory requirements. This contrasted with other subpopulations which lacked satellite telemetry data, creating increased uncertainty in ecological and demographic parameters, skewing survival estimates and making management decisions riskier (Laidre *et al.* 2022). A study on wolverines (*Gulo gulo*) even found higher survival probability for individuals equipped with GPS collars, compared to non-collared individuals, indicating that GPS collars could shield wolverines from poaching (Milleret *et al.* 2021).

Evaluating the species-specific reliability of data collected from marked individuals is crucial for making strong and valid conclusions, especially in long-term studies, since research methods that contravene animal welfare risk not only inhumane treatment of animals but also unreliable findings (Field *et al.* 2019).

In this study, we aimed to evaluate the short- and long-term consequences of capture and surgery on brown bear behaviour and physiology. Our study group consisted of bears that had been captured twice per year, once in winter during their hibernation and again during their active period in summer. They underwent surgery and invasive tissue sampling via muscle biopsies with the aim of utilising them as a translational model for sedentary lifestyle-related diseases in humans. The experimental group (Winter/Summer captures) were compared with a control group that had only been captured once, in spring, as part of ongoing SBBRP work that entailed equipping them with biologgers but without taking muscle biopsies. Firstly, we aimed to evaluate whether bears captured in the winter showed prolonged behavioural effects that persisted into the summer. Secondly, we sought to investigate for how long and to what extent were the movement and body temperature patterns of the same bears, captured in summer, affected. Additionally, we assessed whether bears captured in winter and summer compensated for lost foraging by prolonging their hyperphagic period and delayed the onset of hibernation.

## Materials and methods

### Study area, captures and animals

Our study area was located in a region of south-central Sweden dominated by coniferous forest that consisted mainly of Scots pine (*Pinus sylvestris*) and Norway spruce (*Picea abies*) (Moe *et al.* 2007). We captured 53 solitary brown bears (40 females, 13 males, 75 individual bear years) from 2010–2019 (Table 1) and equipped them with global positioning system (GPS) collars (Vertex Plus, Vectronic Aerospace GmbH, Berlin, Germany), which recorded the bears’ location at hourly intervals. Captures and surgical procedures were carried out in accordance with an established protocol which included darting by helicopter and den captures with medetomidine-tiletamine-zolazepam and additional administration of ketamine when necessary as well as oxygen supplementation for all bears (Arnemo & Evans 2017). All captures and surgical procedures were approved by the Swedish Ethical Committee on Animal research (Uppsala, Sweden; Dnr C3/2016 and Dnr C18/15). Procedures were carried out in the field with as close an adherence to asepsis as possible, including sterile instruments and gloves (Mulcahy 2013) as per the recommendations of Fiorello *et al.* (2016) and further detailed in Arnemo and Evans (2017). We observed three mortalities within 30 days after capture in summer which are therefore classified as capture-related mortalities (Arnemo *et al.* 2006). One two year old female drowned during capture while a second individual (a three year old male) was almost certainly killed by another bear 20 days after capture, as indicated by feeding signs on the deceased bear and the surrounding site when visiting the GPS positions (A Friebe, personal comment 2023). The cause of death for the third bear (a two year old male), which died ten days after capture, remains unknown. Data from the latter two bears were included until the day of presumed death. Bears, captured as offspring of previously marked females, were followed from birth; otherwise age was determined through recourse to the annuli of a cross-section of the premolar roots (Harshyne *et al.* 1998) (Table 1).

**Table 1.** Sample size, bodyweight (kg), age of the bear (years) and day of capture/dummy capture (day of the year) for each individual capture event for bears captured in winter and summer and bears belonging to the control group. Statistics are presented as median (min–max)

Status	N (individual capture events)	Bodyweight (kg)	Age (years)	Day of capture (day of the year)
*Movement*		*Winter/Summer capture*		
Female	37	48 (27–84)	3 (2–4)	163 (156–188)
Male	11	48 (22–77)	2 (2–3)	164 (159–188)
		*Control group*		
Female	22	59 (27–85)	4 (2–4)	162 (155–186)
Male	5	62 (46–76)	3 (3–4)	158 (158–172)
*Body temperature*		*Winter/Summer capture*		
Female	12	44 (28–72)	2 (2–4)	164 (159–188)
Male	3	42 (40–51)	2 (2–3)	182 (160–182)
		*Control group*		
Female	6	60.5 (47.6–74.5)	3.5 (2–5)	164 (157–183)
Male	4	57.8 (39–64)	2	186 (180–192)

Bears that were captured in summer (day of the year [DoY]) 155–192, i.e. June–July) had previously been captured in winter (mid-February) during their hibernation phase in their dens and were categorised as ‘Winter/Summer capture’ (n = 48 individual capture events in summer, for details see Evans *et al.* 2016b and Arnemo & Evans 2017). On both capture occasions, a biopsy (250–350 mg) was taken from the vastus lateralis muscle (located on the lateral side of the thigh) via an open biopsy.

As a control group we used GPS and/or body temperature (T_b_) data of previously captured bears, which fell within the bodyweight and age class range (Table 1) of the bears captured in summer but their previous capture was ≥ 30 days ago, (n = 27, i.e. the bears belonging to the control group were not captured in winter or summer but the previous spring after emergence from the den). For all control group bears we randomly assigned a dummy capture date within the same range and from the same distribution of capture dates of those captured in summer (DoY 155–192, see Supplementary material, Figure S1). The average time difference between the control group bears’ previous capture and their dummy capture event in summer was 56 (± 8) days, which is longer than bears have been shown to be impacted by a capture event (Cattet *et al.* 2008). The individuals from the control group therefore provided an estimate about the bears’ assumed normal movement and body temperature patterns during that time of the year (for examples of raw movement and T_b_ data, see Supplementary material, Figure S2 A–C).

Additionally, in 27 out of 48 captures in summer, bears also underwent abdominal surgery to either have a very high frequency (VHF) transmitter implant (Telonics Inc, Mesa, AZ, USA, serial number IMP–400–2, weight 95 g) or ATS Inc (Isanti, MN, USA, serial number M1250B, weight 100 g) or temperature logger (DST Centi, Star Oddi, Iceland) inserted or removed from the abdominal cavity as previously described (Arnemo & Evans 2017). All implants were sterilised with ethylene oxide gas (Anaprolene AN74i 80 L, Andersen Europe, Kortrijk, Belgium) prior to implantation. In one summer capture event an abdominal surgical procedure was performed without muscle biopsy. Additionally, a total of 19 individuals were equipped with an intra-abdominal temperature logger (DST Centi) to record T_b_ at 5-min intervals. Loggers were inserted on the day of capture for the bears captured in summer and 46–449 days prior to the dummy capture date for the control group bears. Prior to surgery, an analgesic, meloxicam (Metacam®, Boehringer Ingelheim Vetmedica GmBH, Germany) was administered at a dose of 0.2–0.4 mg kg^–1^, as it is recommended for any sampling procedure that requires penetration of the skin by a tool larger than a hypodermic needle (Chinnadurai *et al.* 2016).

During handling and the surgical procedure, the bears’ physiological parameters and reflexes were monitored and measures applied to avoid hypo- or hyperthermia (Arnemo & Evans 2017). In case of hypothermia, i.e. T_b_ ≥ 2°C below normal (Kreeger *et al.* 2023), the bears were moved into the sun and/or covered with an insulated blanket and an external heat source applied. Hyperthermia, i.e. T_b_ ≥ 2°C above normal (Kreeger *et al.* 2023), was treated by removing the animal from direct sunlight, spraying or packing the animal’s groin and feet with cold water and water bags and intravenous administration of Ringer’s solution (Kreeger *et al.* 2023). All captured bears in winter and summer were administered 0.5 L Ringer® acetate intravenously to avoid hyperthermia, but also to compensate for fluid loss due to the chase by the helicopter (in summer) as well as the blood samples that are taken as part of the translational medicine study (average of 200 ml of blood taken from each individual bear).

After the process, including biopsy, surgical procedure and sampling, was finished, bears were returned to the den they had previously occupied during the winter or left in the shade at the capture location or at a location within their home range during the summer captures and the reversal drug administered.

### Statistical analysis

All data cleaning and statistical analyses were performed in R (version 4.2.1). We used the R package *mgcv* and built three sets of generalised additive mixed models (GAMMs) (Wood 2012) with median hourly distance moved per day and hourly mean body temperature as response variables (see Supplementary material, Model framework). The movement metric ‘Median hourly distance moved per day’ refers to the distance (m) the bear moved per hour when active, averaged per day. We applied a gamma family distribution with a log-link function for the movement models and a gaussian distribution for the T_b_ models. Hourly distance moved was calculated as the Euclidean distance a bear moved between consecutive successful GPS positions with the R package *adehabitatLT* (Calenge 2011). Relocations of < 50 m h^–1^ were considered inactive positions and only active positions (≥ 50 m h^–1^) were included in the movement analysis (Bogdanović *et al.* 2021). Differences in movement patterns and physiology were accounted for by including the sex and bodyweight of the individual bears as an interaction in all models. Furthermore, we added a random intercept for the individual bear and capture date and an autoregressive model structure (AR1) for both the movement and T_b_ models to account for temporal autocorrelation in the data.

#### Effects of winter captures (baseline movement model)

The first model was set up with median hourly distance moved per day as the response variable and included the day of the year and the capture category as an interaction (Supplementary material, Model framework). This model helped to identify any potential differences in baseline movement rates from the beginning of April until the end of October between bears which were captured in winter and the following summer and those from the control group.

#### Effects of summer captures

Furthermore, we aimed to identify how long and to what extend the movement and body temperature patterns of the bears were affected by the capture event in summer and set up a list of candidate models for movement and T_b_ as response variables focusing on the time periods preceding and following the capture event (Supplementary material, Model framework).

##### Time after capture

We included a continuous ‘Time since capture’ variable with days as time unit for the movement analyses and hours for the T_b_ analyses. Based on previous literature on the potential effect of capture on movement and physiology, we modelled movement for 90 days and T_b_ for 720 h (i.e. 30 days) post-capture (Cattet *et al.* 2008; Evans *et al.* 2016b). The ‘Time since capture’ variable was included as an interaction with capture category (Winter/Summer capture vs Control group) in both movement and T_b_ models.

We also included a model with a variable that made a further distinction between bears that underwent both a muscle biopsy and abdominal surgery at the same capture event and those only undergoing a muscle biopsy without an abdominal surgery (surgery category). This variable was included as an interaction with ‘Time since capture’ in the movement analysis but not in the T_b_ analysis, since all individuals but one underwent both a biopsy and abdominal surgery (Supplementary material, Model framework).

Additionally, we built one null model and one model with DoY instead of the ‘Time since capture’ interaction to evaluate whether patterns in movement and T_b_ were driven instead by seasonal variation (see Supplementary material, Model framework). Hour of the day was included to control for diurnal T_b_ cycles in the T_b_ models. We considered movement and T_b_ to differ significantly between the two capture categories if the 95% simultaneous confidence interval (CI) of the modelled difference did not overlap with zero (van Rij *et al.* 2015). We used Aikake Information criterion (AIC) to select the highest ranked model within ΔAIC ≤ 2 (Bolker 2022).

##### Time before capture

Moreover, we modelled movement and T_b_ 30 days prior to the capture event by replacing the ‘Time since capture’ variable with ‘Time before capture’ but otherwise keeping the same model structures as used in the highest ranked models for the time after capture to quantify baseline differences between bears captured in winter and summer and the control group (Supplementary material, Model framework).

#### Hibernation entry

In addition, DoY of den entry was determined for each individual by applying a piecewise logistic regression in a Bayesian framework (Lindeløv 2020) on the mean daily distance moved from September to December. DoY of den entry was determined as the change point between two segments which are separated by a change in intercept and variance of the daily mean distance moved, i.e. with the first segment representing the bears’ pre-denning movement and the second the start of the denning period associated with a sharp drop in movement rate. A *t*-test was deployed to test for differences between den entry of bears that were captured in summer and bears of the control group (for more information on models, see the Supplementary Material, Den entry).

## Results

**Figure 1.**
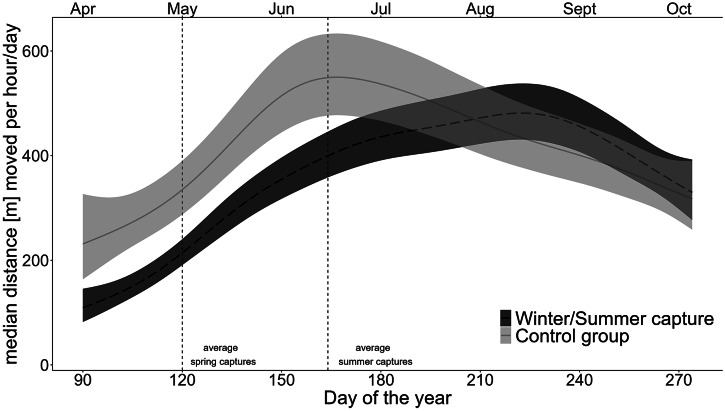
Predictions of the Generalised additive mixed model for median hourly movement rate per day (m) of bears captured in winter and summer (dark grey dashed line) and bears belonging to the control group (light grey solid line) from the beginning of April until the end of October. Predictions were standardised for a solitary female brown bear, weighing 50 kg in Sweden. The shaded areas represent the 95% confidence interval (CI). The vertical dashed lines present the average day of the year of the captures conducted in spring (control group) and of the captures conducted in summer.

Bears captured in winter and summer showed reduced movement rates from the beginning of April (post den exit phase) until the beginning of August, compared to the control group (Figure 1).

When looking at the effects of the captures in summer, for both parameters, movement and T_b_, the model with the interaction of ‘Time since capture’ and the capture category (Winter/Summer capture vs Control group) was the highest ranked model (Table 2).

**Table 2.** AIC model selection table for movement and body temperature models after a summer capture event

Model specification	dAIC	dLogLik	df	weight
*Movement*				
s(DsC, by = capture category)	0.0	124.5	74.8	0.95
s(DsC, by = surgery category)	5.9	125.1	78.3	0.05
s(DoY)	72.0	87.2	73.4	0.00
Null model	239.9	0.0	70.2	0.00
*Body temperature*				
s(TsA_hours, by = capture category)	0.0	1839.5	46.0	1.00
s(DoY)	537.6	1566.7	42.0	0.00
Null model	3638.7	0	25.9	0.00

All movement models additionally included an interaction term of body size and sex as well as a random intercept for the individual bear at the capture event and therefore only varied in the terms specified in the table below. All body temperature models additionally included an interaction term of body size and sex, a smoother term of hour of the day and a random intercept for the individual bear at the capture event. The prefix ‘s’ indicates that the term was included as a smoother to account for non-linearity. dAIC = delta AIC, logLik = log-likelihood, df = degrees of freedom, weight = AIC weight. DsC = Days since capture, capture category = 2 levels (Winter/Summer capture vs Control group), DoY = day of the year, TsA_hours = Time since Antidot (h), surgery category = 3 levels (Only muscle biopsy vs Muscle biopsy + abdominal surgery vs Control group).

**Figure 2.**
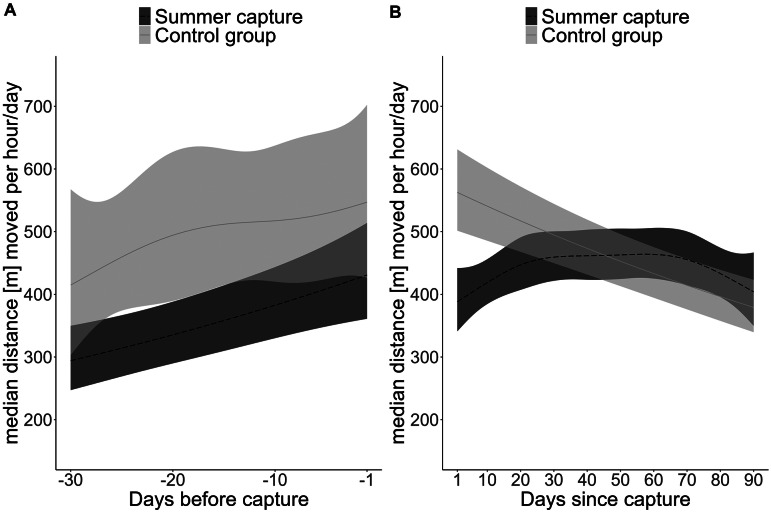
Predictions of Generalised additive mixed models for median hourly movement rate per day (m) for (a) the 60 days preceding and (b) 90 days following a capture event in summer for a solitary female brown bear, weighing 50 kg in Sweden. Dashed (captured bears in summer) and solid (control group) lines represent the mean predicted movement and T_b_ and the shaded areas represent the 95% confidence interval (CI). The two capture categories were considered significantly different from each other when the 95% CI of the modelled differences did not overlap with zero, which can also be interpreted as when the predicted CI from one capture category does not overlap with the predicted mean of the other capture category.

The predicted hourly movement of bears which were going to be captured in summer, i.e. had been previously captured in winter, was already significantly lower (29–22%, 121–118 m h^–1^) 30–4 days prior to the capture event, compared to the control group (Figure 2[a]).

On day one after the capture event, bears captured in summer moved, on average, 42.5 m h^–1^ less than one day before the capture, representing an 11% reduction in movement rate and reached average pre-capture movement rate levels of ~ 430 m h^–1^ on day 14 post-capture.

**Figure 3.**
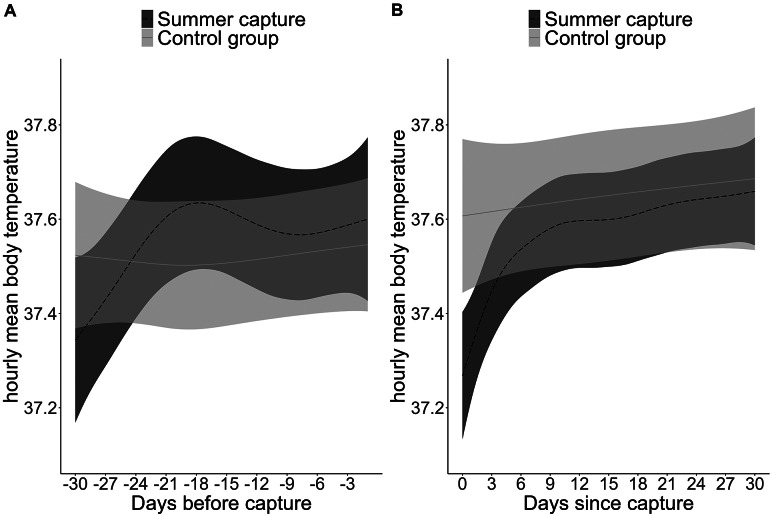
Predictions of Generalised additive mixed models of hourly mean body temperature (T_b_, °C) for (a) the 30 days preceding and (b) 30 days following a capture event in summer for a solitary female brown bear, weighing 50 kg in Sweden. Dashed (captured bears in summer) and solid (control group) lines represent the mean predicted movement and T_b_ and the shaded areas represent the 95% confidence interval (CI). The two capture categories were considered significantly different from each other when the 95% CI of the modelled differences did not overlap with zero, which can also be interpreted as when the predicted CI from one capture category does not overlap with the predicted mean of the other capture category.

Additionally, the predicted hourly movement rate after a capture event was significantly reduced for the first 23 days, starting at a predicted reduction of 31% per hour (174 m) from day 1 after capture to 11% per hour (58 m) on day 23 after capture compared to movement rate of bears in the control group (Figure 2[b]). The T_b_ of bears to be captured in summer did not differ significantly from that of control bears 30 days prior to the capture/dummy capture event (Figure 3[a]). Bears captured in summer had a predicted hourly mean T_b_ of 37.3°C promptly after the capture event compared to the predicted T_b_ of control group bears of 37.7°C, which represents a reduction of less than 1%. T_b_ was reduced for the first 76 h (three days) after capture before returning to the levels of the control group (Figure 3[b]). None of the bears showed signs of hyperthermia or fever in 30 days following capture. The mean den entry date of captured (n = 25 individual den entries) and control group bears (n = 21 individual den entries) did not differ significantly (mean DoY 296 [± 11] vs 299 [± 11]; *P*-value = 0.41).

## Discussion

Brown bears captured in winter showed prolonged behavioural effects from the time of den exit until the late summer as indicated by consistently lower baseline movement rates compared to the control group (Figure 1). When captured the following summer, the bears’ movement rate was further decreased for at least 14 and 23 days post-capture compared to their own pre-capture movement levels and compared to the control group, respectively.

Disturbance of bears during their hibernation phase, including physical captures, has a variety of negative effects, both on their behaviour and physiology. These include den abandonment post-capture, delayed den emergence, as well as a period of two to three weeks post-capture for the bears to restore their physiological state to their baseline hibernation levels (Hellgren & Vaughan 1989; Evans *et al.* 2016b, 2023). This is energetically costly, as the capture event and subsequent den abandonment make the bears arouse from hibernation and become behaviourally active in search of a new denning location. This increases their metabolic rate to several times their basal hibernation metabolic rate and makes them reach euthermic levels (Karpovich *et al.* 2009; Evans *et al.* 2016b). We suggest that bears, captured in winter, incur considerable energetic costs during hibernation. As a result, particularly when viewed in conjunction with delayed onset of their active phase due to delayed den emergence (Evans *et al.* 2016b, 2023), their energy reserves are diminished compared to those not captured in winter. This is reflected in reduced movement rates post-den emergence (Supplementary material, Figure S1), which could be attributed either to attempts to conserve energy or a deficiency in available energy to move more. Movement rates are further lowered immediately following capture a second time in summer and remain lower than the control group until 23 days after the capture event in summer, coinciding with August and the beginning of the hyperphagia phase. Further decreased movement rates post summer capture may have been caused by potential pain perception following repeated muscle biopsy.

In our study, initial drops in movement rates by 11% post summer capture compared to pre-capture levels and a return to baseline movement period of 14 days were likely caused by a combination of the capture, handling and invasive surgery/muscle biopsy procedures. This is less of a drop than that observed by Cattet *et al.* (2008), who found a 20% reduction in movement rates for black and grizzly bears for 3–6 weeks post-capture. However, Cattet *et al.* (2008) did not differentiate between different capture methods and it is possible that other methods, such as the use of leghold snares, may have more drastic impacts on movement rates post-capture than helicopter captures. However, helicopter captures were also associated with elevated levels of aspartate aminotransferase (AST), a marker for muscle injury in the blood serum (Cattet *et al.* 2008). A previous study on haematological and biochemical variables in the same study population of brown bears in Scandinavia during captures in summer found AST values three times higher than the reference levels for captive grizzly bears (Teare 2013; Græsli *et al.* 2015). Surgery and muscle biopsy not to mention the impact of the dart induced soft tissue trauma and whilst quantifying the exact level of pain being experienced by animals in the wild remains an undoubted challenge (Gittleman 2013), the likelihood is that brown bears experience pain as a result of the capture event and surgery, both in winter and in summer. Conducting similar procedures in domestic animals without adequate analgesia is considered inhumane (Chinnadurai *et al.* 2016).

Untreated pain may manifest itself via a variety of psychological and behavioural reactions in laboratory rodents, including sleeplessness, changes in activity, reduced water and food intake and flattened circadian rhythms (Jirkof 2017). Moreover, experiences beyond nociception, including but not limited to nausea, pruritus, thermal stress and fear can contribute to discomfort or suffering (McMillan 2003). Every bear in our study, both in winter and in summer, was given analgesia in the form of meloxicam, a drug with a half-life of 24 h in dogs (*Canis familiaris*) (Leece *et al.* 2005). Nevertheless, reduced movement for at least 14 days post-capture in summer may be an indication that the pain treatment was perhaps insufficient and other, longer-lasting alternatives should be considered as well as providing local anaesthetics as standard procedure for a multi-modal analgesia approach. Local anaesthetics and non-steroidal anti-inflammatory drugs (NSAIDs) provide pain relief, enhance tissue recovery and reduce inflammation (Hollmann *et al.* 2000), which is especially important during the inflammatory phase of wound healing, which may last for up to four days post-surgery (Artlett 2013). There have been several studies on longer-acting analgesic agents, such as meloxicam (Bourne *et al.* 2010; Bauer *et al.* 2014), bupivacaine (Bourne *et al.* 2010; Lascelles & Kirkby Shaw 2016), tramadol (Bourne *et al.* 2010; Fleming & Burn 2014) and buprenorphine (Bourne *et al.* 2010; Soyka 2020), which show promising results in various bear species in captivity as well as long-tailed macaques (*Macaca fascicularis*), cats (*Felis catus*), dogs and humans. It is worth mentioning however that the pharmacokinetics, therapeutic windows and possible side-effects of these drugs are not tested in bears but ongoing clinical use on bears in captivity supports clinical efficacy.

Insufficient pain management is problematic from an animal welfare perspective but also scientifically and methodologically. Scientific results may be affected through altered behaviour and physiology when these are not taken into account or excluded from the study (Jirkof 2017). Despite the widespread implementation of data censoring in ecological research studies following a capture event, there remains a lack of a precise species- and capture method-specific quantification of the duration of time that should be excluded from the dataset. Here, we demonstrate that bears captured in winter and the following summer exhibit markedly distinct movement patterns when compared to the control group. This brings into question the reliability of using these bears as representatives for the normal movement behaviour of subadult bears during the corresponding post-capture period.

The movement pattern of Scandinavian brown bears follows a seasonal trend, with increasing activity from den exit in spring towards summer, followed by a decrease from summer towards den exit in autumn (Evans *et al.* 2016a; Bogdanović *et al.* 2021; Figure 1). This trend occurs in conjunction with a variety of events, including the mating season from May to the beginning of July, natal dispersal of sub-adults and a fluctuation in food availability (Dahle & Swenson 2003; Zedrosser *et al.* 2007; Steyaert *et al.* 2012; Hertel *et al.* 2016). Brown bears in the study area in south-central Sweden are considered adult when they are ≥ 5 and 6 years old for males and females, respectively, but may already have reached sexual maturity by the age of three (Dahle & Swenson 2003). All the bears included in our study were therefore categorised as sub-adults (Table 1), and some of them could have been already affected by mating behaviour in early summer. One other factor influencing movement rates could be natal dispersal of sub-adults (Zedrosser *et al.* 2007).

From June and July (pre-hyperphagia phase) into August and September (hyperphagia phase), brown bears switch their diet from feeding primarily on forb (e.g. wildflowers) and ants to a variety of berry species (Dahle *et al.* 1998). Consequently, late summer and autumn represent the most crucial period for bears, since berry availability is at its highest and bears maximise their food intake to accumulate fat stores prior to hibernation (Manchi & Swenson 2005; Hertel *et al.* 2016). In Scandinavia, this is accompanied by low movement rates, since bears do not need to move much to find new food resources due to the continuous distribution of berries over the forest floor (Hertel *et al.* 2019). This is also the time when the movement rates of bears, captured in winter and summer, catch up with the movement rates of the control group (Figure 1, 2[b]). Within the hyperphagia phase, the movement rate further decreases from the bilberry (July–August) towards the lingonberry season (September–October). Additionally, Evans *et al.* (2016a) found activity levels in Scandinavian brown bears to be affected by ambient temperature around the den entry phase. Thus, decreasing ambient temperatures in autumn may reduce movement rates, which are furthermore accompanied by a lowered metabolism in order to prepare for hibernation. Thus, decreased movement of the bears captured in summer from potential den exit until day 23 after the capture event in summer may result in a delay of natal dispersal and altered mating behaviour as well as insufficient foraging during that period, which needs to be compensated for. Nevertheless, those bears captured in winter and summer entered the den at around the same time as non-captured bears, which may be an indication that they were likely able to compensate for their reduced movement, i.e. the potential loss of foraging opportunities during the initial days after capture and did not pay foraging costs. This is in accordance with the findings of Rode *et al.* (2014), who found that dependent young, which were captured in spring, attained larger adult body size compared to bears that were not captured during their dependent stage, suggesting that impacts of post-capture changes in activity, movement, and feeding behaviour do not result in diminished body condition or compromised survival (Laidre *et al.* 2022).

Reduced body temperature after capture may be explained by a combination of: (1) cooling down during the surgical procedure; (2) disrupted thermoregulatory capabilities in relation to the anaesthesia; and (3) reduced movement post-capture. All bears with a temperature logger captured in summer also underwent abdominal surgery. Due to the opening of the abdomen during surgery, the body of the bear is at risk of cooling down until closure of the incision.

Additionally, medetomidine has been shown to negatively impact the cardiovascular system, causing hypotension and bradycardia as well as disrupted thermoregulation (Kreeger *et al.* 2023). Nevertheless, these effects are rapidly offset following administration of the antagonist, suggesting a lack of persistence over time. However, medetomidine is the only reversible part of the anaesthetic drug combination that can be reversed since there is no antagonist for tiletamine and reversal of zolazepam in animals immobilised with a high dose of tiletamin-zolazepam (TZ) is not recommended (Arnemo & Evans 2017). Since there are no known cardiopulmonary or thermoregulatory side-effects of TZ, this results in long but safe recoveries (Arnemo & Evans 2017). Thus, the duration of behavioural impacts resulting from lingering drug residues within the animals’ systems remains uncertain and a study on black bears showed the presence of tiletamine and zolazepam in muscle tissue and blood serum up to seven days post-anaesthesia (Ryan *et al.* 2009).

T_b_ could be further affected by the potentially diminished foraging abilities linked to lower movement rates, and consequent reduction in caloric intake, as observed in other species (Yoda *et al.* 2000; Lane *et al.* 2012).The combination of abdominal surgery and long recovery associated with little movement following capture may explain lower T_b_ compared to non-captured bears. However, T_b_ reached the level of non-captured bears after three days, indicating that bears return to homeothermy sooner than to their typical movement rates. There were no indications of fever in any of the bears, suggesting that it is unlikely they were experiencing an infection. However, we cannot rule out this possibility entirely.

We posit that the long-lasting impacts seen here primarily reflect the cumulative effects of the capture event in winter and consequent diminished energy reserves as well as the entire capture process in summer, including anaesthesia, surgical procedures and accompanied pain perception as well as exhaustion from the helicopter chase.

### Animal welfare implications

Continuous evaluation of capture and handling protocols is necessary to refine handling methods, to provide adequate pain management and to assess whether replacing live animals and/or reduction in sample size or samples can be applied. Furthermore, it facilitates the assessment of the role of wildlife research in conservation and management challenges (Laidre *et al.* 2022).

Our results indicate substantial negative effects of capture and invasive sampling on the behaviour and physiology of brown bears following a capture event, which is important information for animal ethics committees responsible for evaluating research applications. We would therefore suggest refining capture protocols, i.e. testing longer-acting analgesics and applying a multimodal analgesia approach in order to reduce the impact on behaviour and physiology and facilitate expedited and smooth recoveries of captured wild animals.

## Conclusion

Although the furthering of scientific knowledge is a commendable objective and can enhance our understanding of human obligations toward animals and wildlife, researchers must consistently evaluate the potential knowledge gained against the possible negative impacts on individual animals. For both scientific and ethical considerations, researchers studying animals in their natural habitats are obligated to implement measures that minimise the infliction of fear, distress, or enduring harm upon individual animals (ASAB Ethical Committee/ABS Animal Care Committee 2023).

The evaluation of the impacts resulting from capture, anaesthesia, invasive sampling, and surgical procedures is therefore an essential aspect of conducting ethical wildlife research, enabling the potential refinement of methods. This evaluation aids ethical committees in assessing future research proposals that involve similar methodologies in the field. Consistent with prior research on various bear species (Cattet *et al.* 2008; Rode *et al.* 2014; Evans *et al.* 2016b), our study has demonstrated significant detrimental effects on behaviour and physiology potentially persisting for several months following a capture event in winter and lasting for several days to weeks in summer, despite following best-practice guidelines for physical capture of wildlife, field-based anaesthesia and surgery and the administration of analgesic medication (Chinnadurai *et al.* 2016; Fiorello *et al.* 2016; Arnemo & Evans 2017; Kreeger *et al.* 2023).

The overall aim of this interdisciplinary study to translate findings from a wild species, the brown bear, into human medical research and to enhance medical advancement may justify the invasiveness of the applied sampling procedures and can be considered the biggest benefit of this study. In fact, Natterson-Horowitz *et al.* (2023) suggest that interdisciplinary collaborations are vital to better understand and prevent modern human pathologies. Nonetheless, the documented long-lasting effects of capture and repeated invasive muscle biopsy on behaviour and physiology can be regarded as significant costs for the animals and should be taken carefully into consideration when formulating future capture and handling protocols for free-ranging brown bears. Similarly, ethical committees responsible for assessing research proposals that involve invasive sampling should take these impacts into account when making decisions concerning such applications or when requesting methodological refinements. Also, they should consider the timing of the invasive procedure in relation to the lifecycle of the species in question.

Additionally, conducting assessments to determine how reliable and representative of normal behaviour and physiology biologging data obtained from marked individuals are, is paramount for ensuring robust, unbiased, and valid scientific conclusions. Therefore, wildlife researchers need to be aware of the length of time and extent to which their study animals are affected by the capture event and are advised to account for lasting effects in behaviour and physiology in their data analysis. Additionally, we need to advocate for the animal’s well-being and ideally for the implementation of the 3Rs principles, even though their application may be a challenge in wildlife research (see Soulsbury *et al.* 2020; Box 1).

We suggest use of a multimodal analgesia approach by administering a combination of local and systemic analgesic agents and investigating longer-lasting analgesics as one potential mitigation measure to help reduce the impact of capture, surgery and muscle biopsy on the behaviour of wild animals, if less-invasive methods are not applicable. Furthermore, researchers working with hibernating species need to bear in mind that hibernation represents a phase of the year in which they may be particularly vulnerable. This consideration should be taken into account when formulating methodologies for future research that involve causing disturbance during this period.

## Supporting information

Thiel et al. supplementary material Thiel et al. supplementary material

## Supplementary material

The supplementary material for this article can be found at http://doi.org/10.1017/awf.2023.95.

## References

[r1] Arnemo JM, Ahlqvist P, Andersen R, Berntsen F, Ericsson G, Odden J, Brunberg S, Segerström P and Swenson JE 2006 Risk of capture-related mortality in large free-ranging mammals: experiences from Scandinavia. Wildlife Biology 12: 109–113. 10.2981/0909-6396(2006)12[109:ROCMIL]2.0.CO;2

[r2] Arnemo JM and Evans A 2017 *Biomedical Protocols for Free-ranging Brown Bears, Wolves, Wolverines and Lynx.* Technical Report. https://www.researchgate.net/publication/316351356_Biomedical_Protocols_for_Free-ranging_Brown_Bears_Wolves_Wolverines_and_Lynx

[r3] Arnemo JM, Evans AL, Fahlman Å, Ahlquist P, Brunberg S, Segerström P, Støen OG, Zedrosser A, Söderberg A, Franzén R and Swenson JE 2013 *Capture-related mortalities in brown bears in Scandinavia 1984-2013: A review of 1,824 captures.* Knoxville, Tennessee, USA

[r4] Arnemo JM, Ytrehus B, Madslien K, Malmsten J, Brunberg S, Segerström P, Evans AL and Swenson JE 2018 Long-term safety of intraperitoneal radio transmitter implants in brown bears (*Ursus arctos*). *Frontiers in Veterinary Science 5* 10.3389/fvets.2018.00252 PMC6196346 30374443

[r5] Artlett CM 2013 Inflammasomes in wound healing and fibrosis. The Journal of Pathology 229: 157–167. 10.1002/path.4116 23023641

[r6] ASAB Ethical Committee/ABS Animal Care Committee 2023 Guidelines for the ethical treatment of nonhuman animals in behavioural research and teaching. Animal Behaviour 195: I–XI. 10.1016/j.anbehav.2022.09.006

[r7] Bauer C, Frost P and Kirschner S 2014 Pharmacokinetics of 3 formulations of meloxicam in cynomolgus macaques (*Macaca fascicularis*). Journal of the American Association for Laboratory Animal Science*:* 53: 502–511. 25255073 PMC4181692

[r8] Bogdanović N, Hertel AG, Zedrosser A, Paunović M, Plećaš M and Ćirović D 2021 Seasonal and diel movement patterns of brown bears in a population in southeastern Europe. *Ecology and Evolution.* 10.1002/ece3.8267 PMC8601923 34824804

[r9] Bolker B 2022 *bbmle: Maximum likelihood estimation. R.* https://cran.r-project.org/web/packages/bbmle/bbmle.pdf

[r10] Bourne DC, Cracknell JM and Bacon HJ 2010 Veterinary issues related to bears (*Ursidae*). International Zoo Yearbook 44: 16–32. 10.1111/j.1748-1090.2009.00097.x

[r11] Calenge C 2011 *Analysis of Animal Movements in R: the adehabitatLT Package.* https://www.semanticscholar.org/paper/Analysis-of-Animal-Movements-in-R-%3A-the-Package-Calenge/43d0ecf7bce30b605ccbaf5ea4017a3cfdca9c02

[r12] Cattet M, Boulanger J, Stenhouse G, Powell RA and Reynolds-Hogland MJ 2008 An evaluation of long-term capture effects in Ursids: Implications for wildlife welfare and research. Journal of Mammalogy 89: 973–990. 10.1644/08-MAMM-A-095.1

[r13] Cattet MRL 2013 Falling through the cracks: Shortcomings in the collaboration between biologists and veterinarians and their consequences for wildlife. ILAR Journal 54: 33–40. 10.1093/ilar/ilt010 23904530

[r14] Chanon S, Chazarin B, Toubhans B, Durand C, Chery I, Robert M, Vieille-Marchiset A, Swenson JE, Zedrosser A, Evans AL, Brunberg S, Arnemo JM, Gauquelin-Koch G, Storey KB, Simon C, Blanc S, Bertile F and Lefai E 2018 Proteolysis inhibition by hibernating bear serum leads to increased protein content in human muscle cells. Scientific Reports 8: 5525. 10.1038/s41598-018-23891-5 29615761 PMC5883044

[r15] Chazarin B, Ziemianin A, Evans AL, Meugnier E, Loizon E, Chery I, Arnemo JM, Swenson JE, Gauquelin-Koch G, Simon C, Blanc S, Lefai E and Bertile F 2019 Limited oxidative stress favors resistance to skeletal muscle atrophy in hibernating brown bears (*Ursus arctos*). Antioxidants 8: 334. 10.3390/antiox8090334 31443506 PMC6770786

[r16] Chinnadurai SK, Strahl-Heldreth D, Fiorello CV and Harms CA 2016 Best practice guidelines for field-based surgery and anaesthesia of free-ranging wildlife I. Anaesthesia and analgesia. Journal of Wildlife Diseases 52: S14–S27. 10.7589/52.2S.S14 26845296

[r17] Dahle B, Sørensen OJ, Wedul EH, Swenson JE and Sandegren F 1998 The diet of brown bears Ursus arctos in central Scandinavia: effect of access to free-ranging domestic sheep *Ovis aries*. Wildlife Biology 4: 147–158. 10.2981/wlb.1998.017

[r18] Dahle B and Swenson JE 2003 Seasonal range size in relation to reproductive strategies in brown bears Ursus arctos. Journal of Animal Ecology 72: 660–667. 10.1046/j.1365-2656.2003.00737.x 30893970

[r19] Ebert T, Painer J, Bergman P, Qureshi AR, Giroud S, Stalder G, Kublickiene K, Göritz F, Vetter S, Bieber C, Fröbert O, Arnemo JM, Zedrosser A, Redtenbacher I, Shiels PG, Johnson RJ and Stenvinkel P 2020 Insights in the regulation of trimetylamine N-oxide production using a comparative biomimetic approach suggest a metabolic switch in hibernating bears. Scientific Reports 10: 20323. 10.1038/s41598-020-76346-1 33230252 PMC7684304

[r20] Evans AL, Fuchs B, Singh NJ, Thiel A, Giroud S, Blanc S, Laske TG, Frobert O, Friebe A, Swenson JE and Arnemo JM 2023 Body mass is associated with hibernation length, body temperature, and heart rate in free-ranging brown bears. Frontiers in Zoology 20: 27. 10.1186/s12983-023-00501-3 37587452 PMC10433566

[r21] Evans AL, Singh NJ, Friebe A, Arnemo JM, Laske TG, Fröbert O, Swenson JE and Blanc S 2016a Drivers of hibernation in the brown bear. Frontiers in Zoology 13: 7. 10.1186/s12983-016-0140-6 26870151 PMC4750243

[r22] Evans AL, Singh NJ, Fuchs B, Blanc S, Friebe A, Laske TG, Frobert O, Swenson JE and Arnemo JM 2016b Physiological reactions to capture in hibernating brown bears. Conservation Physiology 4. 10.1093/conphys/cow061 PMC5156896 27990289

[r23] Field KA, Paquet PC, Artelle K, Proulx G, Brook RK and Darimont CT 2019 Publication reform to safeguard wildlife from researcher harm. PLOS Biology 17: e3000193. 10.1371/journal.pbio.3000193 30973871 PMC6459470

[r24] Fiorello CV, Harms CA, Chinnadurai SK and Strahl-Heldreth D 2016 Best-practice guidelines for field-based surgery and anaesthesia on free-ranging wildlife II. Surgery. *Journal of Wildlife Diseases* 52: S28–S39. 10.7589/52.2S.S28 26845297

[r25] Fleming M and Burn C 2014 Behavioural assessment of dental pain in captive Malayan sun bears (*Helarctos malayanus*). Animal Welfare 23: 131–140. 10.7120/09627286.23.2.131

[r26] Fröbert O, Frøbert AM, Kindberg J, Arnemo JM and Overgaard MT 2020 The brown bear as a translational model for sedentary lifestyle-related diseases. Journal of Internal Medicine 287: 263–270. 10.1111/joim.12983 31595572

[r27] Giroud S, Chery I, Arrivé M, Prost M, Zumsteg J, Heintz D, Evans AL, Gauquelin-Koch G, Arnemo JM, Swenson JE, Lefai E, Bertile F, Simon C and Blanc S 2021 Hibernating brown bears are protected against atherogenic dyslipidemia. Scientific Reports 11: 18723. 10.1038/s41598-021-98085-7 34548543 PMC8455566

[r28] Gittleman JL 2013 Carnivore Behavior, Ecology, and Evolution. Springer Science & Business Media: London, UK.

[r29] Græsli AR, Evans AL, Fahlman Å, Bertelsen MF, Blanc S and Arnemo JM 2015 Seasonal variation in haematological and biochemical variables in free-ranging subadult brown bears (*Ursus arctos*) in Sweden. BMC Veterinary Research 11: 301. 10.1186/s12917-015-0615-2 26646442 PMC4673763

[r30] Hackam DG and Redelmeier DA 2006 Translation of research evidence from animals to humans. Journal of the American Medical Association 296: 1727–1732. 10.1001/jama.296.14.1731 17032985

[r31] Harshyne WA, Diefenbach DR, Alt GL and Matson GM 1998 Analysis of error from cementum-annuli age estimates of known-age Pennsylvania black bears. The Journal of Wildlife Management 62: 1281–1291. 10.2307/3801992

[r32] Hellgren EC and Vaughan MR 1989 Denning ecology of black bears in a Southeastern wetland. The Journal of Wildlife Management 53: 347–353. 10.2307/3801136

[r33] Hertel AG, Steyaert SMJG, Zedrosser A, Mysterud A, Lodberg-Holm HK, Gelink HW, Kindberg J and Swenson JE 2016 Bears and berries: species-specific selective foraging on a patchily distributed food resource in a human-altered landscape. Behavioral Ecology and Sociobiology 70: 831–842. 10.1007/s00265-016-2106-2 27217612 PMC4859851

[r34] Hertel AG, Zedrosser A, Kindberg J, Langvall O and Swenson JE 2019 Fluctuating mast production does not drive Scandinavian brown bear behavior. The Journal of Wildlife Management 83: 657–668. 10.1002/jwmg.21619

[r35] Hollmann MW, Durieux ME and Fisher DM 2000 Local anesthetics and the inflammatory response: A new therapeutic indication? Anesthesiology 93: 858–875. 10.1097/00000542-200009000-00038 10969322

[r36] Jirkof P 2017 Side effects of pain and analgesia in animal experimentation. Lab Animal 46: 123–128. 10.1038/laban.1216 28328895

[r37] Karpovich SA, Tøien Ø, Buck CL and Barnes BM 2009 Energetics of arousal episodes in hibernating arctic ground squirrels. Journal of Comparative Physiology. B, Biochemical, Systemic, and Environmental Physiology 179: 691–700. 10.1007/s00360-009-0350-8 19277682

[r38] Kreeger T, Arnemo J, Caulkett N, Hampton J and Meyer L 2023 Handbook of Wildlife Chemical Immobilization, Sixth Edition.Terry Kreeger: Pretoria, South Africa

[r39] Laidre KL, Durner GM, Lunn NJ, Regehr EV, Atwood TC, Rode KD, Aars J, Routti H, Wiig Ø, Dyck M, Richardson ES, Atkinson S, Belikov S and Stirling I 2022 The role of satellite telemetry data in 21st century conservation of polar bears (*Ursus maritimus*). *Frontiers in Marine Science* 9

[r40] Lane JE, Kruuk LEB, Charmantier A, Murie JO and Dobson FS 2012 Delayed phenology and reduced fitness associated with climate change in a wild hibernator. Nature 489: 554–557. 10.1038/nature11335 22878721

[r41] Lascelles BDX and Kirkby Shaw K 2016 An extended release local anaesthetic: potential for future use in veterinary surgical patients? Veterinary Medicine and Science 2: 229–238. 10.1002/vms3.43 29067198 PMC5645851

[r42] Leece EA, Brearley JC and Harding EF 2005 Comparison of carprofen and meloxicam for 72 hours following ovariohysterectomy in dogs. Veterinary Anaesthesia and Analgesia 32: 184–192. 10.1111/j.1467-2995.2005.00207.x 16008715

[r43] Lindeløv JK 2020 *January 5 mcp: An R Package for Regression With Multiple Change Points. OSF Preprints.* 10.31219/osf.io/fzqxv

[r44] Lindsjö J, Fahlman Å and Törnqvist E 2016 Animal welfare from mouse to moose: Implementing the principles of the 3Rs in wildlife research. Journal of Wildlife Diseases 52: S65–77. 10.7589/52.2S.S65 26845301

[r45] Luu BE, Lefai E, Giroud S, Swenson JE, Chazarin B, Gauquelin‐Koch G, Arnemo JM, Evans AL, Bertile F and Storey KB 2020 MicroRNAs facilitate skeletal muscle maintenance and metabolic suppression in hibernating brown bears. Journal of Cellular Physiology 235: 3984–3993. 10.1002/jcp.29294 31643088 PMC13482664

[r46] Manchi S and Swenson JE 2005 Denning behaviour of Scandinavian brown bears *Ursus arctos*. Wildlife Biology 11: 123–132. 10.2981/0909-6396(2005)11[123:DBOSBB]2.0.CO;2

[r47] McMillan FD 2003 A world of hurts—is pain special? Journal of the American Veterinary Medical Association 223: 183–186. 10.2460/javma.2003.223.183 12875442

[r48] Milleret C, Bischof R, Dupont P, Brøseth H, Odden J and Mattisson J 2021 GPS collars have an apparent positive effect on the survival of a large carnivore. Biology Letters 17: 20210128. 10.1098/rsbl.2021.0128 34186003 PMC8241484

[r49] Moe T, Kindberg J, Jansson I and Swenson J 2007 Importance of dietary behaviour when studying habitat selection: examples from female Scandinavian brown bears (*Ursus arctos*). Canadian Journal of Zoology 85: 518–525. 10.1139/Z07-034

[r50] Mortensen RM and Rosell F 2020 Long-term capture and handling effects on body condition, reproduction and survival in a semi-aquatic mammal. Scientific Reports 10: 17886. 10.1038/s41598-020-74933-w 33087816 PMC7578049

[r51] Mulcahy DM 2013 Legal, ethical, and procedural bases for the use of aseptic techniques to implant electronic devices. Journal of Fish and Wildlife Management 4: 211–219. 10.3996/092012-JFWM-080

[r52] Natterson-Horowitz B, Aktipis A, Fox M, Gluckman PD, Low FM, Mace R, Read A, Turner PE and Blumstein DT 2023 The future of evolutionary medicine: sparking innovation in biomedicine and public health. Frontiers in Science: 0. 10.3389/fsci.2023.997136 PMC10590274 37869257

[r53] Nordli K, Wabakken P, Eriksen A, Sand H, Wikenros C, Maartmann E and Zimmermann B 2023 Spatial and temporal cohesion of parents and offspring in a social large carnivore. Animal Behaviour 197: 155–167. 10.1016/j.anbehav.2022.12.006

[r54] Rode KD, Pagano AM, Bromaghin JF, Atwood TC, Durner GM, Simac KS and Amstrup SC 2014 Effects of capturing and collaring on polar bears: findings from long-term research on the southern Beaufort Sea population. Wildlife Research 41: 311–322. 10.1071/WR13225

[r55] Rosenthal N and Brown S 2007 The mouse ascending: perspectives for human-disease models. Nature Cell Biology 9: 993–999. 10.1038/ncb437 17762889

[r56] Ryan CW, Vaughan MR, Meldrum JB, Duncan RB and Edwards JW 2009 Retention Time of Telazol in Black Bears. The Journal of Wildlife Management 73: 210–213. 10.2193/2008-182

[r57] Saraux C, Le Bohec C, Durant JM, Viblanc VA, Gauthier-Clerc M, Beaune D, Park Y-H, Yoccoz NG, Stenseth NC and Le Maho Y 2011 Reliability of flipper-banded penguins as indicators of climate change. Nature 469: 203–206. 10.1038/nature09630 21228875

[r58] Sikes RS and Gannon WL 2011 Guidelines of the American Society of Mammalogists for the use of wild mammals in research. Journal of Mammalogy 92: 235–253. 10.1644/10-MAMM-F-355.1 PMC5909806 29692469

[r59] Soulsbury CD, Gray HE, Smith LM, Braithwaite V, Cotter SC, Elwood RW, Wilkinson A and Collins LM 2020 The welfare and ethics of research involving wild animals: A primer. Methods in Ecology and Evolution 11: 1164–1181. 10.1111/2041-210X.13435

[r60] Soyka M 2020 Recent advances in the treatment of opioid use disorders. Techniques in Neurosurgery & Neurology 3: 1–5.

[r61] Stenvinkel P, Avesani CM, Gordon LJ, Schalling M and Shiels PG 2021 Biomimetics provides lessons from nature for contemporary ways to improve human health. Journal of Clinical and Translational Science 5: e128. 10.1017/cts.2021.790 34367673 PMC8327543

[r62] Stenvinkel P, Fröbert O, Anderstam B, Palm F, Eriksson M, Bragfors-Helin A-C, Qureshi AR, Larsson T, Friebe A, Zedrosser A, Josefsson J, Svensson M, Sahdo B, Bankir L and Johnson RJ 2013 Metabolic changes in summer active and anuric hibernating free-ranging brown bears (*Ursus arctos*). PLOS ONE 8: e72934. 10.1371/journal.pone.0072934 24039826 PMC3767665

[r63] Steyaert SMJG, Endrestøl A, Hackländer K, Swenson JE and Zedrosser A 2012 The mating system of the brown bear *Ursus arctos*. Mammal Review 42: 12–34. 10.1111/j.1365-2907.2011.00184.x

[r64] Teare JA 2013 ISIS physiological reference intervals for captive wildlife: 913 species partitioned by age and gender for large sample sizes. International Species Information System, Minneapolis, MN, USA.

[r65] Thienel M, Müller-Reif JB, Zhang Z, Ehreiser V, Huth J, Shchurovska K, Kilani B, Schweizer L, Geyer PE, Zwiebel M, Novotny J, Lüsebrink E, Little G, Orban M, Nicolai L, El Nemr S, Titova A, Spannagl M, Kindberg J, Evans AL, Mach O, Vogel M, Tiedt S, Ormanns S, Kessler B, Dueck A, Friebe A, Jørgensen PG, Majzoub-Altweck M, Blutke A, Polzin A, Stark K, Kääb S, Maier D, Gibbins JM, Limper U, Frobert O, Mann M, Massberg S and Petzold T 2023 Immobility-associated thromboprotection is conserved across mammalian species from bear to human. Science 380: 178–187. 10.1126/science.abo5044 37053338

[r66] van Rij J, Wieling M, Baayen RH and van Rijn D 2015 *itsadug: Interpreting Time Series and Autocorrelated Data Using GAMMs.* https://research.rug.nl/en/publications/itsadug-interpreting-time-series-and-autocorrelated-data-using-ga

[r67] Whiteman JP, Harlow HJ, Durner GM, Regehr EV, Amstrup SC, Pagano AM and Ben-David M 2022 The acute physiological response of polar bears to helicopter capture. The Journal of Wildlife Management 86: e22238. 10.1002/jwmg.22238 35915725 PMC9324155

[r68] Wilson RP, Holton M, Wilson VL, Gunner R, Tysse B, Wilson GI, Quintana F, Duarte C and Scantlebury DM 2019 Towards informed metrics for examining the role of human-induced animal responses in tag studies on wild animals. Integrative Zoology 14: 17–29. 10.1111/1749-4877.12328 29851254

[r69] Wilson RP and McMahon CR 2006 Measuring devices on wild animals: what constitutes acceptable practice? Frontiers in Ecology and the Environment 4: 147–154. 10.1890/1540-9295(2006)004[0147:MDOWAW]2.0.CO;2

[r70] Wood S 2012 *mgcv: Mixed GAM Computation Vehicle with GCV/AIC/REML smoothness estimation.* https://researchportal.bath.ac.uk/en/publications/mgcv-mixed-gam-computation-vehicle-with-gcvaicreml-smoothness-est

[r71] Yoda T, Crawshaw LI, Yoshida K, Su L, Hosono T, Shido O, Sakurada S, Fukuda Y and Kanosue K 2000 Effects of food deprivation on daily changes in body temperature and behavioral thermoregulation in rats. American Journal of Physiology-Regulatory, Integrative and Comparative Physiology 278: R134–R139. 10.1152/ajpregu.2000.278.1.R134 10644631

[r72] Zedrosser A, Støen O-G, Sæbø S and Swenson JE 2007 Should I stay or should I go? Natal dispersal in the brown bear. Animal Behaviour 74: 369–376. 10.1016/j.anbehav.2006.09.015

