# Supplementary material for: The cost of research: Lasting effects of capture, surgery and muscle biopsy on brown bear (Ursus arctos) movement and physiology
Source: Anim Welf. 2023 Nov 21;32:e75. doi: 10.1017/awf.2023.95 (PMC10951663; doi:10.1017/awf.2023.95)

Figure S1

Density plots illustration the distributions of capture/dummy capture dates for A) GPS/movement data set and B) body temperature data set.

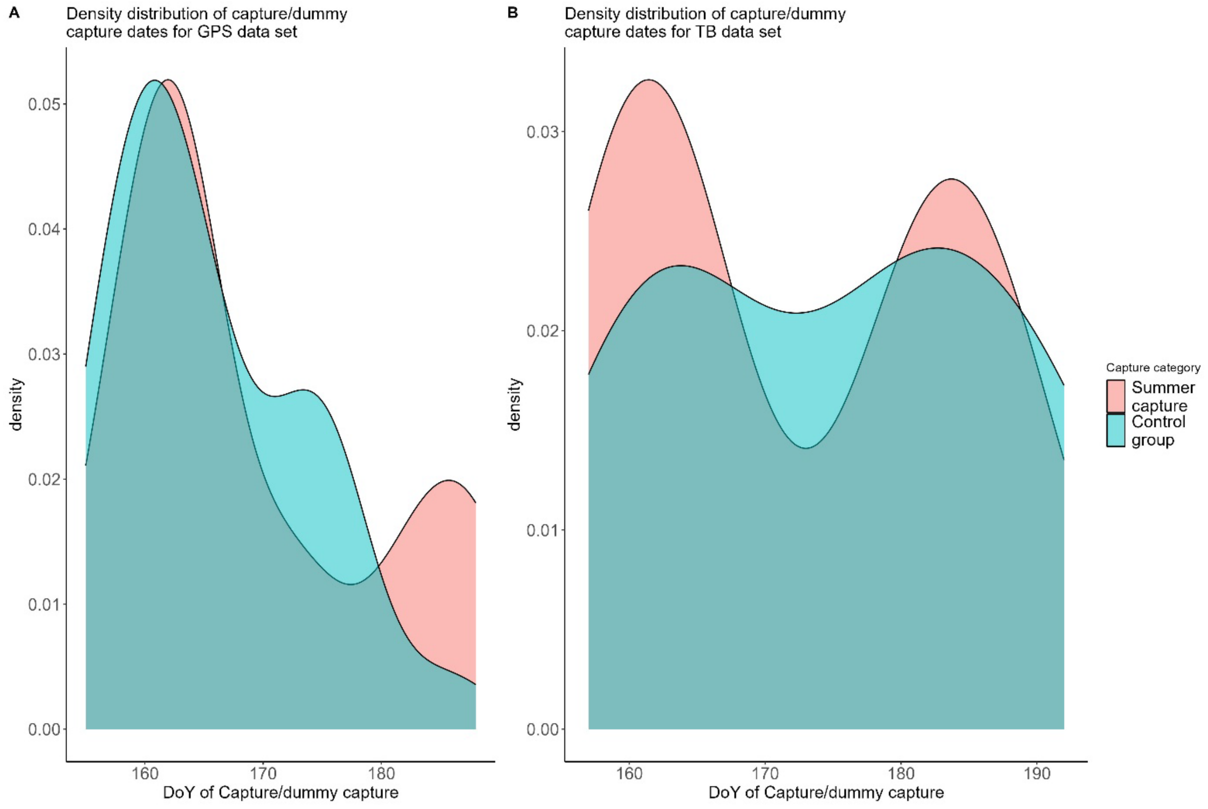

Figure S2

Illustration of raw movement (A and B) and body temperature (C) data of two individuals for A) from the beginning of April (day of the year 90) until end of May (day of the year 150) to illustrate lower movement rates from den exit on, and B) 90 days and C) 30 days after a summer capture event.

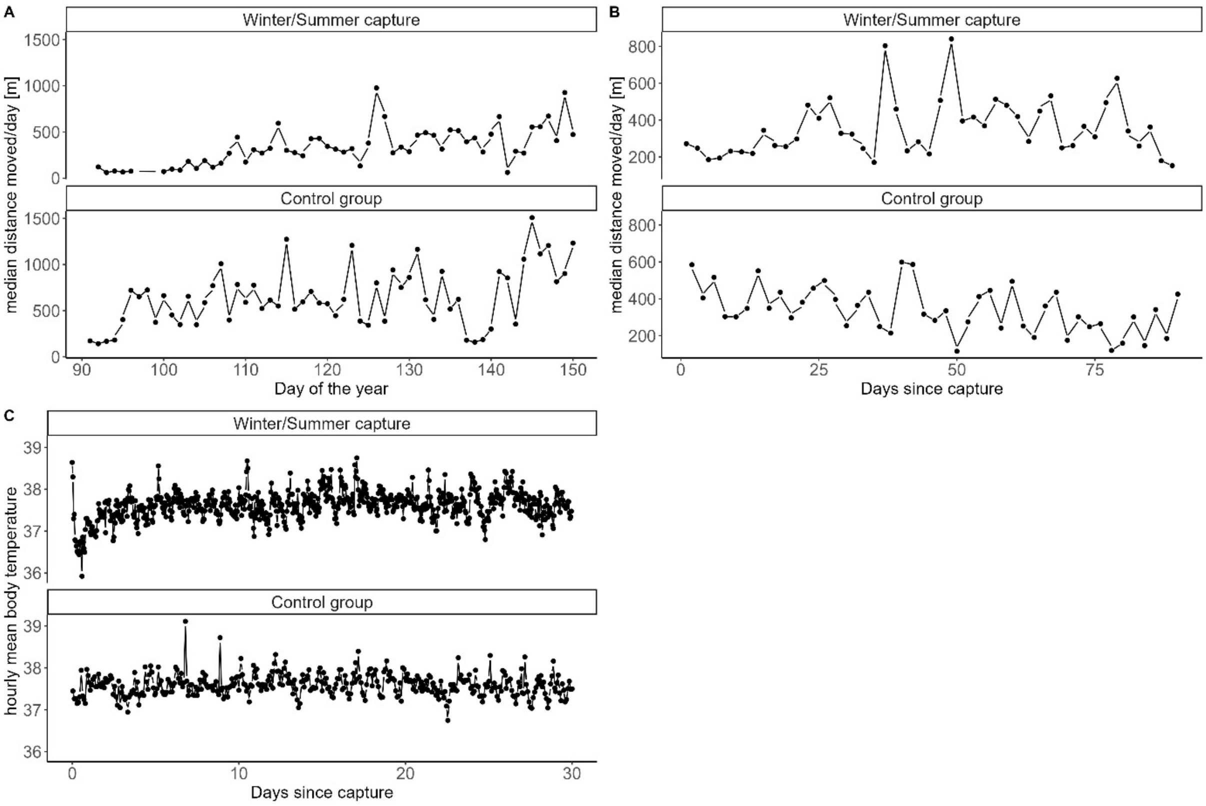

Model framework

Variables:

`median_dist` = response variable for movement models [m], describing how far a bear has moved within one hour if this was considered an active relocation (≥ 50 m/h)

`mean_BodyTemp` = response variable for body temperature models [°C], describing the hourly mean body temperature of the bear

`yday` = day of the year (numeric)

`DsC` = days since/before capture (numeric)

`TsA_hours` = hours since/before capture (numeric)

`Capture` = Capture category (factor, 2 levels = Winter/Summer capture vs. Control group)

`Surgery` = Surgery category (factor, 3 levels = Muscle biopsy + Abdominal surgery vs. Only Muscle biopsy vs. Control group)

`LifeWeight` = body mass of the bear [kg] (numeric)

`Sex` = sex of the bear (factor, 2 levels = Male vs. Female)

`Hour` = hour of the day (numeric, 0 – 23)

`Bear_CaptureDate` = random intercept for individual bear ID and capture date

<u>Baseline model for movement (Effects of winter capture)</u>

```
m <- bam(median_dist ~ s(yday, by = Capture) +
           Capture +
           LifeWeight * Sex +
           s(Bear_CaptureDate, bs = "re"),
         data = GPS,
         family = Gamma(link = "log"),
         AR.start = GPS$Event,
         rho = 0.35,
         discrete = TRUE)
```

<u>Effects of summer captures</u>

<u>*Movement models (after capture)*</u>

```
# NULL MODEL
m0 <- bam(median_dist ~ 1 +
               s(Bear_CaptureDate, bs = "re"),
               family = Gamma(link = "log"),
               data = GPS,
```

```
            control = gam.control(trace = FALSE),
            method = "ML",
          gamma = 1.5)

# MODEL WITH DAY OF THE YEAR TO TEST FOR SEASONAL EFFECT
m1 <- bam(median_dist ~ s(yday, bs = "cr") +
             LifeWeight*Sex +
             s(Bear_CaptureDate, bs = "re"),
             family = Gamma(link = "log"),
             data = GPS,
           control = gam.control(trace = FALSE),
           method = "ML",
          gamma = 1.5)

# MODEL WITH CAPTURE CATEGORY
m2 <- bam(median_dist ~ s(DsC, by = Capture, bs = "cr") +
             Capture +
             LifeWeight*Sex +
             s(Bear_CaptureDate, bs = "re"),
             family = Gamma(link = "log"),
             data = GPS,
           control = gam.control(trace = FALSE),
           method = "ML",
          gamma = 1.5)

# MODEL WITH SURGERY CATEGORY
m3 <- bam(median_dist ~ s(DsC, by = Surgery, bs = "cr") +
             Surgery +
             LifeWeight*Sex +
             s(Bear_CaptureDate, bs = "re"),
             family = Gamma(link = "log"),
             data = GPS,
           control = gam.control(trace = FALSE),
           method = "ML",
          gamma = 1.5)
```

The highest ranked model was refitted with an autoregressive model structure (AR1) and the “fREML” method:

```
m2 <- bam(median_dist ~ s(DsC, by = Capture, bs = "cr") +
                               Capture +
                               LifeWeight*Sex +
                              s(Bear_CaptureDate, bs = "re"),
              family = Gamma(link = "log"),
              data = GPS,
            control = gam.control(trace = FALSE),
            method = "fREML",
           gamma = 1.5,
         AR.start = GPS$Event,
         rho = 0.35,
         discrete = TRUE)
```

<u>Movement model (before capture)</u>

```
mB <- bam(dist ~ s(DsC, by = Capture) +
           Capture +
           LifeWeight * Sex +
           s(Bear_CaptureDate, bs = "re"),
         data = GPS_BEFORE,
         family = Gamma(link = "log"),
         AR.start = GPS_BEFORE$Event,
         rho = 0.35,
         discrete = TRUE,
         method = "fREML")
```

Body temperature models (before capture)

```
# NULL MODEL
m0 <- bam(meanBodyTemp ~ 1 +
                      s(Bear_CaptureDate, bs = "re"),
               family = gaussian(link = "identity"),
               data = TB,
             control = gam.control(trace = FALSE),
             method = "ML",
           gamma = 1.5)

# MODEL WITH DAY OF THE YEAR TO TEST FOR A SEASONAL PATTERn
m1 <- bam(meanBodyTemp ~ s(yday, bs = "cr") +
                     s(Hour, bs = "cc") +
                     LifeWeight*Sex+
                     s(Bear_CaptureDate, bs = "re"),
              family = gaussian(link = "identity"),
              data = TB,
            control = gam.control(trace = FALSE),
            method = "ML",
          gamma = 1.5)

# MODEL WITH CAPTURE CATEGORY
m2 <- bam(meanBodyTemp ~ s(TsA_hours, by = Capture, bs = "cr")  +
                     Capture +
                     LifeWeight*Sex+
                     s(Hour, bs = "cc") +
                     s(Bear_CaptureDate, bs = "re"),
              family = gaussian(link = "identity"),
              data = TB,
            control = gam.control(trace = FALSE),
            method = "ML",
            gamma = 1.5)
```

The highest ranked model was refitted with an autoregressive model structure (AR1) and the “REML” method:

```
m2ac <- bam(meanBodyTemp ~ s(TsA_hours, by = Capture, bs ="cr") +
                      Capture +
                      LifeWeight*Sex+
                      s(Hour, bs = "cc", k = 15) +
                      s(Bear_CaptureDate Date, bs = "re"),
               family = gaussian(link = "identity"),
               data = TB,
             control = gam.control(trace = FALSE),
             method = "REML",
             AR.start = TB$Event,
             rho = 0.7,
             gamma = 1.5)
```

__Body temperature model (after capture)__

```
mB <- bam(meanBodyTemp ~ s(TsA_hours, by = Capture, bs = "cr")  +
                     Capture +
                     LifeWeight*Sex+
                     s(Hour, bs = "cc") +
                     s(Bear_CaptureDate, bs = "re"),
              family = gaussian(link = "identity"),
              data = TB_Before,
            control = gam.control(trace = FALSE),
            method = "REML",
            gamma = 0.7,
             AR.start = TB_Before$Event,
             rho = 0.75)
```

Den Entry

Model specification

```
model<-list(
  mean_dist~1,
  ~1+sigma(1)
)
```

Model

```
fit<-mcp(model, x1[, c("mean_dist", "yday")],
          par_x="yday")
```

Plot example

```
plot(fit, q_fit = T)+
  ggtitle("Determination of den entry based on mean daily distance moved")+
  xlab("Day of the year")+ylab("Mean daily distance moved")+
  theme_bw()
```

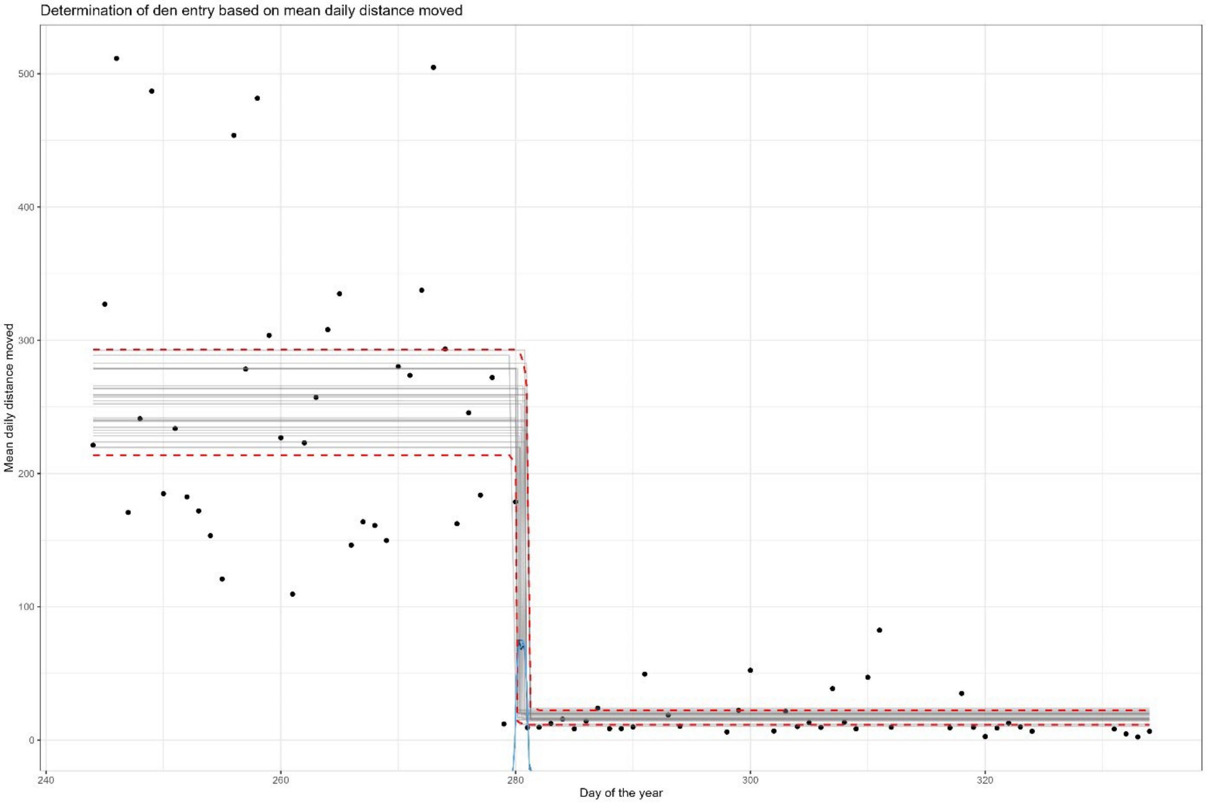

Supplement: Thiel et al. supplementary material [file S0962728623000957sup001.pdf]
